# Supplementary material for: Polyketide synthase gene clusters in the ericoid mycorrhizal fungus Oidiodendron maius: genome-wide prediction, expression profiling, and functional characterization of knockout mutants
Source: Front Plant Sci. 2026 Sep 16;17:1924430. doi: 10.3389/fpls.2026.1924430 (PMC13623903; doi:10.3389/fpls.2026.1924430)
Supplement: Supplementary file 1 [file SupplementaryFile1.zip › Supplementary Materials/Supplementary Folder 1.zip!/Oidma1_AssemblyScaffolds_Repeatmasked/knownclusterblast/cluster59/jgi.p_Oidma1_107109_mibig_hits.html]

| MIBiG Protein | Description | MIBiG Cluster | MiBiG Product | % ID | % Coverage | BLAST Score | E-value |
| --- | --- | --- | --- | --- | --- | --- | --- |
| EHA28236.1 | monooxygenase | BGC0001143 | Azanigerone\_biosynthetic\_gene\_cluster | 39 | 90.0203665988 | 332 | 8e-91 |
| AAS90062.1 | CypX | BGC0000009 | Aflatoxin\_biosynthetic\_gene\_cluster | 34 | 97.1486761711 | 264 | 3.5e-70 |
| AAS90036.1 | CypX | BGC0000008 | Aflatoxin\_biosynthetic\_gene\_cluster | 34 | 100.0 | 261 | 1.7e-69 |
| AAS90107.1 | CypX | BGC0000006 | Aflatoxin\_biosynthetic\_gene\_cluster | 34 | 100.0 | 260 | 5e-69 |
| BAE71332.1 | cytochrome\_P450\_monooxygenase | BGC0000004 | Aflatoxin\_biosynthetic\_gene\_cluster | 34 | 100.0 | 259 | 1.1e-68 |
| AAS90082.1 | CypX | BGC0000010 | Aflatoxin\_biosynthetic\_gene\_cluster | 34 | 100.0 | 256 | 5.6e-68 |
| AAC49196.1 | putative\_p450\_monooxygenase | BGC0000152 | Sterigmatocystin\_biosynthetic\_gene\_cluster | 30 | 93.482688391 | 188 | 2.4e-47 |
| Bcin12g06380.1 |  | BGC0000631 | Botrydial\_biosynthetic\_gene\_cluster | 25 | 96.9450101833 | 178 | 1.9e-44 |
| ALA62324.1 | methylphloroacetophenone\_synthase | BGC0001309\_c2 | Usnic\_acid\_biosynthetic\_gene\_cluster | 29 | 86.9653767821 | 170 | 4e-42 |
| ADZ25011.1 | cytochrome\_P450\_hydroxylase | BGC0000380 | Leupyrrin\_biosynthetic\_gene\_cluster | 32 | 45.6211812627 | 99 | 8.7e-21 |
| ADD83003.1 | PtnO2 | BGC0001156 | Platencin\_biosynthetic\_gene\_cluster | 28 | 36.0488798371 | 86 | 1.3e-16 |
| ACO31275.1 | PtmO2 | BGC0001140 | Platensimycin\_/\_platencin\_biosynthetic\_gene\_cluster | 29 | 36.0488798371 | 84 | 2.9e-16 |
